# Is Cyclosporine Ototoxic?

**DOI:** 10.3389/fneur.2020.593917

**Published:** 2020-10-22

**Authors:** Sofia Waissbluth

**Affiliations:** Department of Otolaryngology, Pontificia Universidad Católica de Chile, Santiago, Chile

**Keywords:** cyclosporine, immunosuppressant drug, hearing loss, ototoxicity, dizziness, transplant, nephrotic syndrome

## Introduction

Cyclosporine was discovered in 1970, it was isolated from the fungus *Tolypocladium inflatum* in Switzerland (1). Soon after, an immunosuppressive property was suggested, and by 1978, it was used for the first time in human kidney transplantation to treat organ rejection post-transplant (2). It is now used as a treatment for solid organ, as well as bone marrow transplantation. Its immunomodulatory capacity has extended its use to other immune-mediated diseases such as severe forms of rheumatoid arthritis and psoriasis, steroid-dependent, frequently relapsing or steroid-resistant nephrotic syndrome, graft vs. host disease, refractory posterior uveitis/Behçet disease and amyotrophic lateral sclerosis (2, 3).

## How Does Cyclosporine Work as an Immunosuppressant?

Cyclosporine suppresses T cell activation, by inhibiting calcineurin, as a result of binding with cyclophilin A. Consequently, calcineurin cannot dephosphorylate nuclear factor of activated T cell (NFAT) and therefore inhibits the activation of genes required for proliferation such as IL-2, IL-4, as well as CD40L (4). It has also been demonstrated that cyclosporine can also block the activation of JNK and p38 signaling pathways involved in T cell activation (5, 6). Inhibition of these pathways are the main mechanisms of cyclosporine's immunosuppressive effects.

Cyclosporine also has an effect on the innate immune cells including dendritic cells, macrophages and neutrophils. Dendritic cells are essential in linking the innate and adaptive immune response, and it is believed that cyclosporine can modulate cytokine production in these cells and lead to altered induction of T cell responses (7).

Cyclosporine is an inhibitor of the P-glycoprotein multidrug efflux pump, also known as Multidrug Resistance Protein 1 (MDR1). It is a competitive inhibitor of MDR1. As cyclosporine is lipophilic, not only is it a substrate for MDR1 (8), but it can also enter cells by passive diffusion (3). MDR1 is expressed in several tissues, primarily epithelial cells with excretory functions (9). It is widely expressed by innate and adaptive immune cells; macrophages, dendritic cells, naive B cells, CD8+ T cells, CD4+ T cells, and NK cells (8).

## What Are the Known Side Effects of Cyclosporine?

While cyclosporine is now a commonly used immunosuppressant, its use is not without side effects. Nephrotoxicity, hypertension, neurotoxicity, hepatotoxicity, hirsutism, gingival hyperplasia, lymphoproliferative neoplasms, opportunistic infections, hyperuricemia, hyperkalemia, and hypomagnesemia are some of the adverse effects described (10–12).

The drug is erratically absorbed from the gastrointestinal tract which leads to variability in blood concentrations and unpredictable pharmacokinetics (10, 13). Cyclosporine also has a narrow therapeutic window, which means that minimal changes in dosage may lead to therapeutic failures, or adverse effects (14). Therefore, blood levels must be carefully monitored during treatments with this drug (13). Cyclosporine neurotoxicity can occur at normal or elevated drug levels. It has been described that the majority of neurological side effects involve the central nervous system, however, peripheral nerves can be affected. Autopsy results have shown ischemic lesions, neuronal loss, hemorrhagic foci and diffuse neuronal damage (11).

It has been suggested that cyclosporine, in combination with methylprednisolone, can cause microangiopathy (15) and ischemic lesions by endothelial damage and vasoconstriction (11).

## Why Would Cyclosporine Cause Ototoxicity?

Cyclosporine is a calcineurin inhibitor, and calcineurin has been detected in the cochlear nerve and inner hair cells of rats (16). We know cyclosporine is an immunosuppressant, and although the cochlea is not considered an immunological organ, it has been shown to constitutively express a variety of immune-related molecules involved in multiple signaling pathways. A better understanding of the functional roles of cochlear immune cells with regards to cochlear homeostasis and disease formation is needed (17). Cyclosporine could perhaps impact immune activities within the cochlea.

Hearing loss can result from ischemic events in the cochlea (18), and peripheral vestibulopathy can also result from vascular processes (19). Also, it is known that cyclosporine can cause hypomagnesemia, and magnesium appears to be an important cation in the auditory system. Magnesium deficiency has been linked to noise-induced hearing loss, ototoxicity, and sudden sensorineural hearing loss (SSNHL) (20, 21). The exact mechanism by which magnesium deficiency can cause hearing loss is not fully understood (22).

Another possible contributing factor is the fact that cyclosporine is a lipophilic molecule (23), and lipophilic drugs have greater diffusion through the blood labyrinthine barrier (BLB) (24). This could be a potential pathway for cyclosporine reaching inner ear cells. Another point to consider here is that presence of perivascular resident macrophages and pericytes on the vessels of the stria vascularis. The role of these pericytes is unknown, but we do know that pericytes in other organs (i.e., kidney, lung, liver, retina) play a role in regulating capillary blood flow and endothelial activity. Also, it is believed that the perivascular resident macrophages could play a role in regulating barrier integrity (25). Hence, these new classes of cells of the BLB could play a role here.

As a result of these observations, it is therefore reasonable to investigate whether cyclosporine can in fact cause hearing loss or vestibular symptoms. Hence, microangiopathy, neurotoxicity and/or hypomagnesemia may play a role in cyclosporine induced hearing loss or vestibulopathy.

## Studies Reporting Hearing and Vestibular Symptoms

Various studies have aimed to assess hearing loss or vestibular symptoms with regards to cyclosporine treatment (Table 1). Ibrahim et al. performed audiometry testing for children with idiopathic nephrotic syndrome treated with cyclosporine (26). They observed that 23.7% (*n* = 19/80) of the patients had hearing loss, however, 47% had sensorineural hearing loss (SNHL) and 53% had conductive hearing loss (CHL). Between the children with and without hearing loss, there was no significant difference in the median cyclosporine treatment duration. Of note, 31% (*n* = 25/80) of the patients had also received cyclophosphamide, and the authors report that cyclophosphamide intake was significantly greater in patients with CHL vs. SNHL. They suggest this may be due to recurrent ear infections caused by immunosuppression. It is important to remember that the most common cause of pediatric CHL is otitis media with effusion, a highly prevalent condition in children which is generally reversible (33).

**Table 1:** Studies reporting auditory and/or vestibular symptoms with the use of cyclosporine.

**Study**	**Age**	***n***	**CsA treatment**			**Follow-up**	**Hearing loss eval. method**	**Hearing loss**	**Tinnitus**	Vestibular symptoms^*^	**Other drugs used**
			**Indication**	**Duration**	**Dosage**						
Ibrahim et al. (26)	9.7 ± 3 yrs (5–16)	80	Idiopathic nephrotic syndrome	17 m (2–24)	–	At least 12 m	Pure tone audiometry	23.7% (19/80)	–	–	Cyclophosphamide, methylprednisolone
Kasap-Demir et al. (27)	129.06 ± 63.25 m	16	Nephrotic syndrome (SDNS, FRNS, SRNS)	40.22 ± 35.14 (7–129) m	Cumulative: 4,480.9 ± 3,652.86 (630–13,466) mg/kg	30.44 ± 19.88 m (1–53)	Pure tone audiometry, TEOAE	0%	0%	–	Steroids, rituximab, cyclophosphamide, levamisole, mycophenolate mofetil, angiotensin converting enzyme inhibitors
Gulleroglu et al. (28)	14.05 ± 4.11 yrs	27	Renal transplant	29.8 ± 22.5 m	–	21.3 ± 12.2 m	Pure tone audiometry, questionnaire	63% (17/27)	4% (1/27)	0%	Mycophenolate mofetil, prednisolone
Fortes et al. (29)	46.2 yrs	18	Liver transplant	–	–	636 days (65–939)	Pure tone audiometry	–	–	–	Prednisone, azathioprine, vancomycin, nystatin, sulfamethoxazole-trimethoprim
Rifai et al. (30)	51 ± 17 yrs	478	Orthotopic liver transplant	–	–	8 ± 5 yrs	Questionnaire	15% (73/478)	11% (54/478)	–	Tacrolimus, mycophenolate mofetil, prednisolone, sirolimus, azathioprine
Groothoff et al. (31)	29.3 yrs (20.7–41.7)	106	Renal transplant	–	–	11.3 yrs (0–29.9)	Questionnaire	8%	–	–	Prednisone, azathioprine, tacrolimus, antihypertensive (various)
Cole et al. (32)	46 yrs	1,097	Renal transplant	18 m	- CsA: 257 ± 97 mg/day - CsA-ME: 248 ± 80 mg/day	18 m	?	Overall: 4.7% (52/1,097)			Prednisone, azathioprine

*CsA, cyclosporine; CsA-ME, cyclosporine microemulsion; m, months; yrs, years; eval, evaluation; SDNS, steroid-dependent nephrotic syndrome; FRNS, frequently-relapsing nephrotic syndrome; SRNS, steroid-resistant nephrotic syndrome; TEOAE, transient evoked otoacoustic emissions*.

* *dizziness, vertigo. Parenthesis represent range*.

Kasap-Demir et al. also evaluated hearing loss in pediatric patients with nephrotic syndrome treated with cyclosporine for at least 6 months. They report that none of the patients had hearing loss, conductive or sensorineural (27). The patients were also exposed to multiple other drugs including cyclophosphamide, steroids, levamisole, rituximab, mycophenolate mofetil and antihypertensive (angiotensin-converting enzyme inhibitors).

Gulleroglu et al. evaluated the prevalence of hearing loss in renal transplant patients, they report that 63% of patients had SNHL, and 1 patient had tinnitus. Interestingly, they report two cases of SSNHL, an uncommon occurrence in pediatric otolaryngology. The patients with hearing loss were 17 years old (±4.4). They also observed that second-hour serum cyclosporine levels were significantly higher in patients with hearing loss and/or decreased speech understanding when compared to the group without hearing impairment. No vertigo was reported. They suggest that the hearing impairment following transplantation may be a dose-dependent cyclosporine toxicity (28).

Fortes et al. evaluated adult patients undergoing liver transplantation with a hearing test, before and after transplantation. Patients received either cyclosporine or tacrolimus (FK-506) as part of their immunosuppressive regimen. For the cyclosporine group, the time elapsed between transplantation and the post-transplant hearing test was 635.83 days (range 65–939). No clinically significant changes were seen on pure tone audiometry. They do, however, report statistically significant changes with the use of tacrolimus (29).

Rifai et al. sent out a questionnaire to liver transplantation recipients, with a mean follow-up period of 8 ± 5 years since the transplant. Excluding patients with a prior history of hearing loss, they observed that 15% reported hearing loss, and 11%, tinnitus. Of interest, the mean time period from transplantation to onset of hearing impairment was 4 ± 4 years. Self-reported hearing loss was considered severe in 62%, and sudden hearing loss was the cause of hearing impairment in 26% of these patients. Hearing loss was associated with the use of tacrolimus immunosuppression, but not with cyclosporine (30).

Groothoff et al. performed a cross sectional investigation of patients who underwent renal transplantation. While their cohort is quite large, with 249 subjects, the cross-sectional analysis was completed for patients who were alive, with a functioning graft, and for which health and social status could be obtained. Of 106 patients that answered the questionnaire, 8% reported hearing problems, and 69.2% have used cyclosporine as part of their treatment at some point in time. There was no association between hearing problems and the use of cyclosporine (31).

Finally, Cole et al. compared long-term safety and tolerability of cyclosporine and cyclosporine microemulsion for adult renal transplant patients. As previously mentioned, cyclosporine pharmacokinetics can be tricky, and the microemulsion formulation was created in order to obtain a more consistent drug exposure, showing a significant increase in the area under the concentration-time curve and maximum plasma concentration. By 18 months, they observed that overall, 4.7% had hearing loss. This was more common in the microemulsion group (cyclosporine: 2.8%, *n* = 10/356; microemulsion: 5.7%, *n* = 42/737). Mild and moderate hearing and vestibular disorders were more frequent in the microemulsion group (32).

## Case Reports

To the best of my knowledge, four case reports have been published with regards to hearing impairment due to cyclosporine treatment. Arinsoy et al. reported the case of a 22-year-old male patient that underwent a renal transplant. Cyclosporine treatment began before the surgery and was maintained, he also received methylprednisolone and azathioprine. On post-op day seven, the patient presented with a unilateral SSNHL, with a flat curve on the audiogram, and 12% speech discrimination. No dizziness was described. It was believed to be a vascular event, and was treated with vasodilator and antiaggregant therapy. Seven days later, he had regained his hearing. Initially, his blood levels of cyclosporine were at 200 ng/ml, the dosage was decreased, and by the time he had regained his hearing, blood levels were at 50.8 ng/ml (34). Porges et al. describe the case of a 22-year-old male patient with severe Crohn's disease that received treatment with cyclosporine. On the fifth day of treatment, the patient exhibited neurotoxicity and a gastrointestinal bleed requiring blood transfusions. His cyclosporine blood level was elevated (1,290 ng/ml). They mention bilateral asymmetric SNHL detected by audiometry, but no details are provided (35). Marioni et al. reported the case of a 32-year-old male patient who underwent a renal transplant 7 years prior. He was treated with cyclosporine, azathioprine and methylprednisolone. He then developed a progressive bilateral SNHL. No dizziness was described. Cyclosporine dosage was decreased, and 32-weeks later, hearing loss remained stable, and did not worsen. Interestingly, this patient was only taking cyclosporine and methylprednisolone at the time of hearing loss onset (36). Finally, Tafazoli et al. published their experience with a 26-year-old female patient receiving cyclosporine for graft vs. host disease. An accidental overdose occurred with the microemulsion formulation, and the patient developed nausea, vomiting, flushing, chest tightness, tremor and vertigo. The neurological examination revealed a benign paroxysmal positional vertigo (37).

## Discussion

The literature regarding the ototoxic potential of cyclosporine is lacking. The published studies include patients with nephrotic syndrome (26, 27), renal transplantation (28, 31, 32) and liver transplantation (29, 30). These patients usually receive a variety of drugs, and their underlying condition is a confounding factor. For instance, Saha et al. observed that children with idiopathic nephrotic syndrome were at greater risk for developing hearing loss. They suggest that high cumulative doses of furosemide and hypocalcemia could be risk factors (38). Also, it is known that patients with chronic diseases such as renal failure on hemodialysis are at greater risk of hearing loss (39). These patients could have received furosemide at some point during their treatment, a loop diuretic, known to be ototoxic (40). Many of the patients described in the included studies and case reports have received various drugs, including methylprednisolone, a steroid hormone that can increase cyclosporine blood levels. Many drugs including certain antibiotics, calcium channel blockers and steroids can increase blood levels of cyclosporine as they are cytochrome P-450 inhibitors, and cyclosporine is metabolized in the liver (41).

The opposite is true as well, cyclosporine can alter tissue distribution of P-glycoprotein substrates. As an example, Saito et al. showed that mice receiving doxorubicin did not develop hearing loss as it is extruded by the P-glycoprotein multidrug efflux pump from the inner ear. Yet, when given in combination with cyclosporine, cochlear damage and dysfunction of the auditory pathway was seen, resulting from a significant accumulation of doxorubicin in the inner ear (42). On the other hand, Yang et al. used a transtympanic injection of cyclosporine in a guinea pig model of sterile labyrinthitis to evaluate whether it suppresses inner ear inflammation and hearing loss. While cyclosporine was not effective in preventing hearing loss, it did not worsen it either (43).

Cyclosporine presents intra and inter-individual variability in blood concentrations and unpredictable pharmacokinetics (10, 13). It has a narrow therapeutic window (14) and can interact with multiple drugs (41). While there are reports of hearing loss and vertigo relating to its use, too many variables including medical condition treated, concomitant treatments, electrolyte imbalance, dosage, blood levels, cytochrome P-450 metabolism, duration of treatment and pre-existing hearing impairment limit me in being able to state that cyclosporine is in fact an ototoxic medication. Further studies are necessary to determine, with some level of certainty, whether cyclosporine is ototoxic or not, and to assess if microangiopathy, neurotoxicity or hypomagnesemia play a role.

Literature is currently lacking with regard to risk factors for developing hearing loss when receiving a treatment with cyclosporine. For instance, the synergistic effect of impaired renal function. Most of the patients included in the reviewed studies had some degree of renal insufficiency, or even underwent renal transplantation. Other known ototoxic drugs are known to affect the kidney as well, causing nephrotoxicity, such as cisplatin and aminoglycosides (44, 45). We know that there are similarities between the kidney and inner ear cells. For instance, the basement membranes of the stria vascularis and glomerular area are comparable (46), and we also know of various oto-renal syndromes such as branchio-oto-renal syndrome, Townes-Brocks syndrome, Alport syndrome, among others (47).

Because of the lack of evidence, I cannot elaborate further on a potential ototoxicity monitoring program, however, clinicians dealing with patients receiving cyclosporine as part of their treatment regimen should be alert to the possibility of ototoxicity and screen their patients for hearing loss accordingly. And also, try to avoid the use of a combination of ototoxic medications in these patients.

While cyclosporine is not a commonly used drug in all medical specialties, it is an important drug in transplantation, and in 2019, close to 40,000 patients underwent organ transplantation in the US (48), and most of the people on the waiting list need a kidney or liver (49). There are many unknowns that remain to be studied, regarding cyclosporine pharmacokinetics in the inner ear, whether cells in the BLB play a role and how other drugs such as methylprednisolone may interact with cyclosporine at the cochlear level. I hope this article will provide the scientific community with some enthusiasm to tackle this issue.

## Author Contributions

The author confirms being the sole contributor of this work and has approved it for publication.

## Conflict of Interest

The author declares that the research was conducted in the absence of any commercial or financial relationships that could be construed as a potential conflict of interest.
